# Serum TNF‐α Modulation and Serum TNF‐α Modulation and Functional Recovery Following Taurine Supplementation in Acute Spinal Cord Injury: A Prospective Observational Cohort Study With Healthy Controls

**DOI:** 10.1155/ijin/3637413

**Published:** 2026-10-31

**Authors:** Zeenat Ara, Shah Waliullah

**Affiliations:** ^1^ Department of Orthopaedic Surgery King George’s Medical University Lucknow Uttar Pradesh India kgmu.org

## Abstract

**Background:**

Acute spinal cord injury (SCI) is associated with persistent inflammation and impaired functional recovery. We investigated longitudinal changes in serum tumour necrosis factor‐α (TNF‐α), their association with functional independence, and the relationship of taurine supplementation with inflammatory and functional outcomes after acute traumatic SCI.

**Methods:**

In this prospective observational cohort study, 70 adults with acute traumatic SCI and 70 age‐ and sex‐matched healthy controls were studied at King George’s Medical University, Lucknow, India. Patients received standard management, with 36 receiving oral taurine (3 g/day) and 34 receiving conventional treatment for 6 months; allocation was nonrandomised. Serum TNF‐α was measured at baseline and 6 months, and functional independence was assessed using the Spinal Cord Independence Measure (SCIM). Longitudinal associations were examined using correlation and repeated‐measures correlation analyses, with false discovery rate adjustment. Receiver operating characteristic (ROC) analysis assessed the diagnostic discrimination of baseline TNF‐α, and multivariable linear regression identified independent predictors.

**Findings:**

Serum TNF‐α decreased from 0.7636 (SD 0.5433) pg/mL at baseline to 0.4913 (0.4942) pg/mL at 6 months in the overall SCI cohort (*p* < 0.0001). The decrease was greater in the taurine group (0.7002 [0.5059]–0.2109 [0.0883] pg/mL) than in the conventional‐treatment group (0.8308 [0.5803]–0.7883 [0.5708] pg/mL). At 6 months, TNF‐α in the taurine group was comparable with healthy controls (*p* = 0.2553), whereas concentrations remained elevated in the conventional‐treatment group (*p* < 0.0001). Baseline TNF‐α discriminated SCI from healthy controls with an AUC of 0.892 (95% CI 0.829–0.946; *p* < 0.0001). At 6 months, total SCIM was higher in the taurine group than in the conventional‐treatment group (71.14 [9.78] vs. 34.53 [4.76]; *p* < 0.001), with higher scores across all SCIM domains. Higher TNF‐α was consistently associated with lower functional independence; repeated‐measures correlations were −0.673 for total SCIM, −0.684 for self‐care, −0.642 for respiration and sphincter management and −0.681 for mobility (all FDR‐adjusted *p* < 0.001). However, no significant TNF‐α × time × treatment interaction was observed for total SCIM or any SCIM domain. In multivariable analysis, baseline TNF‐α independently predicted 6‐month TNF‐α (*β* = 0.582, 95% CI 0.409–0.756; standardised *β* = 0.640; FDR‐adjusted *p* < 0.001; *R* ^2^ = 0.494).

**Interpretation:**

Lower TNF‐α concentrations were associated with greater functional independence over 6 months after acute traumatic SCI. Taurine supplementation was associated with greater TNF‐α reduction and higher functional scores, but nonrandomised treatment allocation and the absence of a significant longitudinal treatment interaction preclude causal inference. These findings support TNF‐α as a candidate inflammatory biomarker and justify adequately powered randomised trials to determine the therapeutic efficacy of taurine after SCI.

**Trial Registration:** Clinical Trial Registry of India: CTRI/2022/11/047262

## 1. Introduction

Spinal cord injury (SCI) is a heterogeneous neurological disorder in which disruption of spinal cord integrity produces variable motor, sensory, autonomic and sphincter dysfunction. It is broadly classified as traumatic spinal cord injury (TSCI), resulting from external mechanical forces such as motor vehicle collisions and falls, or nontraumatic spinal cord injury (NTSCI), arising from intrinsic pathological processes including neoplasia, haemorrhage, infection, inflammation and degenerative disease. Clinical severity and functional outcome are governed primarily by the neurological level, extent and completeness of cord damage [1].

Among the secondary injury mechanisms, inflammation plays a critical role in determining the extent of tissue damage and neurological recovery after SCI. Traumatic injury activates resident microglia and promotes infiltration of peripheral immune cells, resulting in the release of numerous proinflammatory mediators. Tumour necrosis factor‐alpha (TNF‐α), a key proinflammatory cytokine, contributes to neuronal apoptosis, demyelination, disruption of the blood–spinal cord barrier and axonal degeneration. Elevated TNF‐α levels have been associated with greater injury severity and poorer neurological outcomes, suggesting that TNF‐α may serve as a useful biomarker for monitoring inflammatory activity and predicting recovery following SCI [2].

Following SCI, inflammatory mediators such as TNF‐α, IL‐1β and IL‐6 are markedly dysregulated during the acute and subacute phases, contributing to a sustained neuroinflammatory milieu that may influence secondary tissue injury and neurological recovery. Accordingly, the use of circulating inflammatory biomarkers to characterise injury severity and predict subsequent neurological trajectories has emerged as an important area of SCI research over the past decade. Biomarker‐based profiling may provide objective molecular correlates of disease severity, refine prognostic assessment and enable biological monitoring of therapeutic response. Nevertheless, clinical evidence linking inflammatory biomarker dynamics with neurological severity and recovery remains limited, particularly in Indian populations [3, 4].

Recent clinical evidence has also highlighted the potential importance of taurine in SCI. Ara et al. [5] reported significantly reduced serum taurine levels in patients with acute traumatic SCI compared with healthy controls in a case–control study with prospective follow‐up. Although serum taurine levels increased during recovery, they remained significantly lower than those of healthy individuals at 6 months. The study further demonstrated good diagnostic performance of serum taurine in distinguishing SCI patients from healthy controls and identified injury severity as an important predictor of taurine levels. These findings suggest that taurine may be involved in SCI pathophysiology and support its potential utility as a biomarker of injury severity and recovery [5].

Taurine (β‐aminoethanesulfonic acid) is a nonproteinogenic amino sulfonic acid with broad cytoprotective and immunomodulatory activity. Beyond its roles in cellular homeostasis and redox regulation, taurine can restrain inflammatory signalling by modulating NF‐κB activation and downstream proinflammatory cytokine production, particularly TNF‐α, IL‐1β and IL‐6. This convergence on inflammatory signalling provides a mechanistic rationale for investigating taurine–TNF‐α interactions in acute tissue injury and neuroinflammation [6].

Despite growing experimental evidence supporting the neuroprotective effects of taurine, clinical studies evaluating its association with inflammatory biomarkers and functional recovery in SCI patients remain scarce. Furthermore, the relationship between changes in serum TNF‐α levels and functional outcomes following taurine supplementation has not been adequately investigated. A better understanding of this relationship may provide valuable insights into the role of inflammation in SCI recovery and help identify potential adjunctive therapeutic strategies.

Therefore, the present study was undertaken to evaluate serum TNF‐α levels in patients with acute SCI and healthy controls, to investigate the association of taurine supplementation with TNF‐α modulation over time, and to examine the relationship between serum TNF‐α levels and functional recovery assessed using the Spinal Cord Independence Measure (SCIM). In addition, receiver operating characteristic (ROC) curve analysis was performed to assess the ability of serum TNF‐α to discriminate patients with SCI from healthy individuals.

## 2. Methods

### 2.1. Study Design

A Prospective Observational Cohort Study with Healthy Controls.

### 2.2. Setting

This prospective observational cohort study was conducted in the Department of Orthopaedic Surgery at King George’s Medical University, Lucknow, India, after obtaining approval from the Institutional Ethics Committee (Ref. Code No. 104th ECM IIB‐Ph.D/P3). The study was conducted in accordance with the Declaration of Helsinki. Written informed consent was obtained from all participants prior to enrolment.

### 2.3. Participants

Sample size was estimated using a two‐tailed hypothesis test with a significance level of 5% (*α* = 0.05) and statistical power of 80% (1 − *β* = 0.80). In the absence of robust published data regarding the magnitude of difference in serum TNF‐α levels between acute SCI patients and healthy controls in the study population, a medium standardised effect size (Cohen’s *d* = 0.48) was assumed. Based on these assumptions, the minimum required sample size was calculated to be 68 participants per group (136 participants in total). To account for potential attrition and incomplete data, the sample size was increased to 140 participants. Accordingly, a total of 140 participants were enrolled, comprising 70 patients with acute SCI and 70 age‐ and sex‐matched healthy controls.

#### 2.3.1. Inclusion Criteria

Patients fulfilling the following criteria were included:

• Age between 18 and 65 years
• Either gender
• Acute traumatic SCI presenting within 1 week of injury
• Complete SCI (ASIA Grade A) or incomplete SCI (ASIA Grades B–D)
• Thoracolumbar Injury Classification and Severity Score (TLICS) ≥ 4 requiring surgical stabilisation
• Provision of written informed consent

#### 2.3.2. Exclusion Criteria

Patients were excluded if they had

• Nontraumatic SCI
• Associated major injuries requiring urgent surgical intervention (severe chest, abdominal or head injuries)
• Severe traumatic brain injury
• Pre‐existing psychiatric illness
• Use of immunosuppressive medications such as corticosteroids
• Medical comorbidities contraindicate surgery.
• Refusal to participate

### 2.4. Group Allocation

Allocation of patients with acute SCI to the taurine supplementation and conventional treatment groups was nonrandomised. The study was designed as a prospective observational study with longitudinal follow‐up rather than as a randomised controlled trial; therefore, no randomisation sequence or allocation concealment procedure was used. Taurine supplementation was offered as an adjunct to standard clinical management, and treatment allocation reflected the patient’s willingness to receive taurine supplementation together with routine clinical management decisions.

All patients received standard treatment according to institutional clinical practice. Patients who elected to receive taurine supplementation received oral taurine in addition to standard treatment, whereas those who did not receive supplementation continued with conventional treatment alone. Because treatment assignment was not randomised, differences between groups in baseline characteristics, injury severity, socioeconomic circumstances, rehabilitation exposure, nutritional status and other prognostic factors could not be completely excluded.

Accordingly, the comparison between treatment groups was considered observational, and the findings were interpreted as associations rather than definitive evidence of a causal treatment effect. The potential for selection bias and residual confounding was therefore explicitly considered in the interpretation of the results.

*Study Groups:* This was a prospective observational cohort study with 70 patients with acute traumatic SCI and 70 age‐ and sex‐matched healthy controls. Healthy controls were recruited from attendants and volunteers without a history of SCI, neurological disease, chronic inflammatory disorders, or other major systemic illnesses. The SCI cohort was further divided into two treatment groups during the study period. Thirty‐six patients received oral taurine supplementation in addition to standard treatment, whereas 34 patients received conventional treatment alone. Patients in both treatment groups included complete (ASIA Grade A) and incomplete (ASIA Grades B–D) spinal cord injuries.

*Group 1 (SCI Patients Receiving Taurine Supplementation):* Patients with acute traumatic SCI who received standard treatment along with oral taurine supplementation (3 g/day) for 6 months.

*Group 2 (SCI Patients Receiving Conventional Treatment):* Patients with acute traumatic SCI who received standard treatment without taurine supplementation.

*Group 3 (Healthy Controls):* Age‐ and sex‐matched healthy individuals who served as controls for comparison of serum TNF‐α levels.

All participants were followed prospectively for 6 months. Serum TNF‐α levels were measured at baseline and at 6 months. Functional outcomes were assessed using the SCIM.

Taurine was administered at a dose of 3 g/day (1000 mg three times daily after meals) for 6 months. Taurine tablets were supplied by Biolife Remedies, Mohali, India. The dose was selected based on previous human clinical studies in non‐SCI populations in which taurine was administered at 3 g/day and was generally well tolerated, including studies in individuals with metabolic disorders and overweight or obesity. A human risk assessment has also identified 3 g/day as an observed safe supplemental intake level for healthy adults based on available clinical trial data. To our knowledge, no previous human clinical trial has evaluated taurine supplementation in patients with SCI; therefore, no SCI‐specific taurine dose has been established. The 3 g/day regimen was consequently selected as a pragmatic dose supported by the existing human taurine‐supplementation literature and administered in three divided doses after meals.

### 2.5. Data Collection and Measurement

At enrolment, baseline characteristics—including demographics, illness history and relevant clinical data—were recorded using a predesigned proforma. Participants received reminder calls 1 week before appointments. At the 6‐month follow‐up, returned medication bottles were collected to count remaining tablets. Participants consuming at least 85% of taurine, calcium and vitamin D3 were considered compliant.

Blood samples were collected twice: shortly after injury and at 6 months. Five millilitres of blood were drawn, allowed to clot for 20 min, then centrifuged at 5000 rpm for 10 min. Serum was stored at −80°C for TNF‐α measurement using a specialised assay kit (catalogue no. abk0601H) according to the manufacturer’s protocol.

### 2.6. Statistical Analysis was Done With SPSS Software (IBM Corp., USA)

Data normality was checked by the Shapiro–Wilk test. Continuous variables were described as mean ± standard deviation or median (interquartile range), as appropriate. Categorical variables were presented as frequencies and percentages. Comparisons of serum TNF‐α levels among taurine‐treated patients, conventional treatment patients and healthy controls were performed using the Kruskal–Wallis test followed by Dunn’s post hoc analysis. Within‐group comparisons between baseline and 6 months were assessed using paired statistical tests as appropriate. Correlation analysis between serum TNF‐α levels and SCIM scores was performed using Pearson’s or Spearman’s correlation coefficients, depending on data distribution.

Furthermore, ROC curve analysis was performed to evaluate the diagnostic performance of serum TNF‐α in differentiating SCI patients from healthy controls. A *p* value of < 0.05 was considered statistically significant.

## 3. Results

### 3.1. Demographic and Clinical Characteristics of Study Participants: Baseline Characteristics

Seventy patients with acute traumatic SCI were included, comprising 36 patients in the taurine group and 34 in the conventional‐treatment group. The mean age of the study population was 32.67 ± 11.00 years (range, 18–55 years), and 57 (81.4%) patients were male. Most participants were from rural areas (52 [74.3%]) and were nonvegetarian (37 [52.9%]). Road traffic accidents (30 [42.9%]) and falls from height (28 [40.0%]) were the predominant mechanisms of injury.

Baseline characteristics of the two treatment groups were broadly comparable. Mean age was 32.69 ± 11.43 years in the taurine group and 32.65 ± 10.68 years in the conventional‐treatment group (*p* = 0.986). The distribution of age categories (*p* = 0.502), sex (*p* = 0.084), marital status (*p* = 0.617), TLISS score (*p* = 0.657), neurological status (*p* = 0.646), AIS grade (*p* = 0.913) and fracture level (*p* = 0.122) did not differ significantly between groups. In both groups, lumbar and thoracolumbar injuries predominated, with AIS grade A being the most frequent neurological grade (Tables 1, 2 and 3).

**TABLE 1:** Baseline and demographic characteristics of the studied cases.

Variable	Category	*n*	%	Mean ± SD
Age (years)	—	—	—	32.67 ± 11.00

Gender	Male	57	81.43	—
	Female	13	18.57	—

Marital status	Married	38	54.29	—
	Unmarried	32	45.71	—

Residence	Rural	52	74.29	—
	Urban	18	25.71	—

Month of injury	January	1	1.43	—
	February	2	2.86	—
	March	8	11.43	—
	April	4	5.71	—
	May	0	0.00	—
	June	2	2.86	—
	July	15	21.43	—
	August	10	14.29	—
	September	6	8.57	—
	October	8	11.43	—
	November	6	8.57	—
	December	8	11.43	—

Food habit	Nonvegetarian	37	52.86	—
	Vegetarian	33	47.14	—

Mode of injury	Road traffic accident	30	42.86	—
	Fall from height	28	40.00	—
	Fall of heavy object	6	8.57	—
	Assault	3	4.29	—
	Fall of wall	1	1.43	—
	Others	2	2.86	—

Fracture vertebrae	Thoracolumbar	44	62.86	—
	Lumbar	25	35.71	—
	Mixed	1	1.43	—

Education	Illiterate	32	45.71	—
	High school	12	17.14	—
	Graduate	14	20.00	—
	Others	12	17.14	—

Occupation	Labour	30	42.86	—
	Student	15	21.43	—
	Driver	9	12.86	—
	Farmer	8	11.43	—
	Housewife	6	8.57	—
	Others	2	2.86	—

**TABLE 2:** Baseline age characteristics of participants in the taurine and conventional‐treatment groups.

Characteristic	Taurine group (*n* = 36)	Conventional‐treatment group (*n* = 34)	Overall (*n* = 70)	*p* value^∗^
Age, years				
Mean (SD)	32.69 (11.43)	32.65 (10.68)	32.67 (11.00)	**0.986**
Median (IQR)	32.00 (22.00–40.25)	29.50 (24.25–37.25)	30.00 (22.25–40.00)	0.860
Range	18–55	18–55	18–55	—
Age category, *n* (%)				**0.502**
18–24 years	12 (33.3)	9 (26.5)	21 (30.0)	
25–39 years	13 (36.1)	17 (50.0)	30 (42.9)	
40–55 years	11 (30.6)	8 (23.5)	19 (27.1)	

*Note:* Values are presented as mean (standard deviation [SD]), median (interquartile range [IQR]), or number (percentage), as appropriate. *p* values compare the taurine and conventional‐treatment groups. Continuous variables were compared using appropriate parametric or nonparametric tests, and categorical variables using the chi‐square or Fisher’s exact test, as appropriate. Statistical significance was defined as *p* < 0.05. Bold values indicate statistically significant results, where applicable.

*p* ^∗^ < 0.05.

*p* ^∗∗^ < 0.01.

*p* ^∗∗∗^ < 0.001.

**TABLE 3:** Baseline demographic and clinical characteristics of the study participants.

Parameter	Taurine group (*n* = 36)	Conventional group (*n* = 34)	*p* value
Sex–male	26 (72.2%)	31 (91.2%)	0.084
Sex–female	10 (27.8%)	3 (8.8%)	
Marital status—single	18 (50.0%)	14 (41.2%)	0.617
Marital status—married	18 (50.0%)	20 (58.8%)	
TLISS score, mean ± SD	6.83 ± 0.94	6.74 ± 0.90	0.657
TLISS score, median (range)	7.0 (5–8)	7.0 (5–8)	
Incomplete paraplegia	21 (58.3%)	17 (50.0%)	0.646
Complete paraplegia	15 (41.7%)	17 (50.0%)	
ASIA Grade A	15 (41.7%)	17 (50.0%)	0.913
ASIA Grade B	8 (22.2%)	7 (20.6%)	
ASIA Grade C	9 (25.0%)	7 (20.6%)	
ASIA Grade D	4 (11.1%)	3 (8.8%)	
Fracture—thoracolumbar	26 (72.2%)	17 (50.0%)	0.122
Fracture—lumbar	10 (27.8%)	16 (47.1%)	
Fracture—lumbar and thoracolumbar	0 (0%)	1 (2.9%)	
Neurological level—lumbar	21 (58.3%)	25 (73.5%)	—
L1	7 (19.4%)	14 (41.2%)	
L2	9 (25.0%)	5 (14.7%)	
L3	3 (8.3%)	6 (17.6%)	
Hypesthesia below L1/L3	2 (5.6%)	0 (0%)	
Neurological level—thoracic/dorsal	15 (41.7%)	9 (26.5%)	—
T6	4 (11.1%)	4 (11.8%)	
D4/T4	2 (5.6%)	3 (8.8%)	
D11/T11	4 (11.1%)	2 (5.9%)	
D6	2 (5.6%)	0 (0%)	
D10	2 (5.6%)	0 (0%)	
T8	1 (2.8%)	0 (0%)	

### 3.2. Serum TNF‐α Concentrations Showed Distinct Longitudinal Trajectories Between Treatment Groups

In the overall cohort, TNF‐α decreased from 0.7636 ± 0.5433 pg/mL at baseline to 0.4913 ± 0.4942 pg/mL at 6 months (*p* < 0.0001), but remained higher than in healthy controls at 6 months (0.2790 ± 0.2256 pg/mL; *p* = 0.0158). In the taurine group, TNF‐α decreased substantially from 0.7002 ± 0.5059–0.2109 ± 0.0883 pg/mL (*p* < 0.0001), with 6‐month concentrations no longer differing significantly from controls (*p* = 0.2553). By contrast, the conventional‐treatment group showed only a modest reduction, from 0.8308 ± 0.5803 to 0.7883 ± 0.5708 pg/mL (*p* < 0.0001), and concentrations remained significantly higher than controls at both time points (both *p* < 0.0001). Thus, taurine supplementation was associated with substantially greater attenuation of TNF‐α, with concentrations approaching those observed in healthy controls by 6 months (Table 4, Figure 1).

**TABLE 4:** Descriptive and comparative statistics of serum TNF‐α concentrations at baseline and 6 months.

Study group/cohort	N	Baseline TNF‐α (pg/mL), mean ± SD [median (IQR)]	6‐month TNF‐α (pg/mL), mean ± SD [median (IQR)]	Within‐group *p* value^†^	*p* value vs. healthy controls, baseline^‡^	*p* value vs. healthy controls, 6 months^‡^
Total patient cohort	70	0.7636 ± 0.5433 [0.4635 (0.3358–1.2424)]	0.4913 ± 0.4942 [0.2855 (0.2162–0.5300)]	< 0.0001	< 0.0001	0.0158
Taurine group (patients 1–36)	36	0.7002 ± 0.5059 [0.4374 (0.3521–1.0496)]	0.2109 ± 0.0883 [0.2251 (0.1326–0.2795)]	< 0.0001	< 0.0001	0.2553
Conventional group (patients 37–70)	34	0.8308 ± 0.5803 [0.5716 (0.3278–1.3206)]	0.7883 ± 0.5708 [0.5525 (0.3038–1.2305)]	< 0.0001	< 0.0001	< 0.0001
Healthy control cohort	70	0.2790 ± 0.2256 [0.2321 (0.2213–0.2684)]	—	—	—	—

Abbreviations: IQR, interquartile range; SD, standard deviation; TNF‐α, tumour necrosis factor‐α.

^†^Within‐group comparisons were assessed using the Wilcoxon signed‐rank test because measurements were longitudinally paired.

^‡^Comparisons with the healthy control cohort were performed using the Mann–Whitney U test.

**FIGURE 1:**
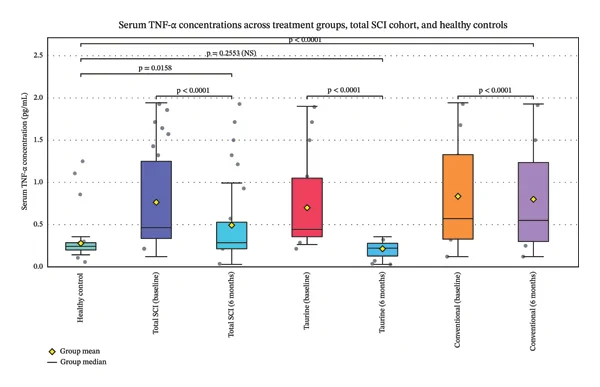
Serum TNF‐α concentration distributions at baseline and 6‐month follow‐up across healthy controls, the total spinal cord injury (SCI) cohort and the taurine and conventional‐treatment groups.

### 3.3. ROC Analysis

ROC analysis showed that baseline serum TNF‐α effectively discriminated patients with SCI from healthy controls. The area under the ROC curve (AUROC) was 0.892 (95% CI, 0.829–0.946; *p* < 0.0001). The optimal TNF‐α threshold was 0.299 pg/mL, corresponding to a sensitivity of 87.14% (61/70) and specificity of 88.57% (62/70), with a Youden index of 0.757. At this threshold, the positive and negative predictive values were 88.41% and 87.32%, respectively, and overall classification accuracy was 87.86% (123/140) (Table 5; Figure 2).

**TABLE 5:** Receiver operating characteristic (ROC) curve of baseline serum TNF‐α for discrimination between patients with acute SCI and healthy controls.

Statistic/metric	Result
SCI cases	70
Healthy controls	70
Total sample	140
AUC	0.892
95% CI for AUC	0.829–0.946
*p* value	< 0.0001
Optimal cutoff	0.299 pg/mL
Youden’s J	0.757
Sensitivity	87.14% (61/70)
Specificity	88.57% (62/70)
Positive predictive value	88.41%
Negative predictive value	87.32%
Overall diagnostic accuracy	87.86% (123/140)

**FIGURE 2:**
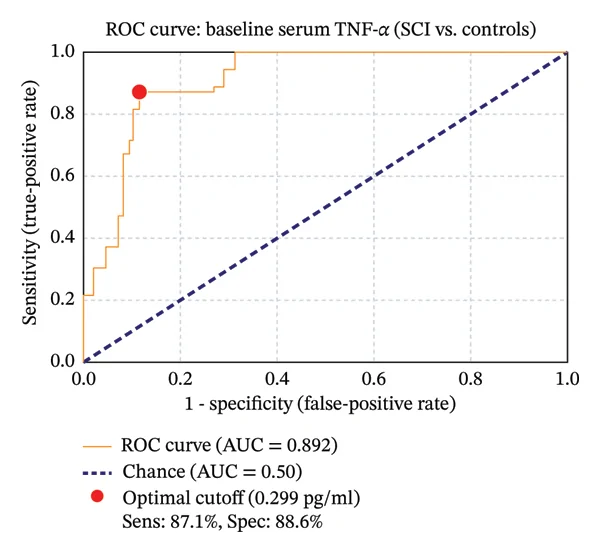
Receiver operating characteristic (ROC) curve analysis of baseline serum TNF‐α for discriminating spinal cord injury (SCI) patients from healthy controls.

ROC analysis showed that TNF‐α moderately discriminated between baseline and 6‐month measurements in the SCI cohort (AUC, 0.731; 95% CI, 0.641–0.817; *p* < 0.0001). The optimal threshold was 0.320 pg/mL, determined using Youden’s J index (*J* = 0.443). At this threshold, TNF‐α demonstrated 80.0% sensitivity and 64.3% specificity, with an overall classification accuracy of 72.1% (101/140 observations). These findings indicate a statistically significant but moderate ability of TNF‐α to distinguish baseline from 6‐month SCI measurements (Table 6, Figure 3).

**TABLE 6:** ROC analysis of TNF‐α for discrimination between baseline and 6‐month measurements in the SCI cohort.

ROC parameter	Result
Sample size	140 (70 baseline; 70 at 6 months)
Area under the curve (AUC)	0.731
95% CI for AUC	0.641–0.817
*p* value	< 0.0001
Optimal TNF‐α cutoff	0.320 pg/mL
Youden’s J index	0.443
Sensitivity	80.0% (56/70)
Specificity	64.3% (45/70)
Overall classification accuracy	72.1% (101/140)

**FIGURE 3:**
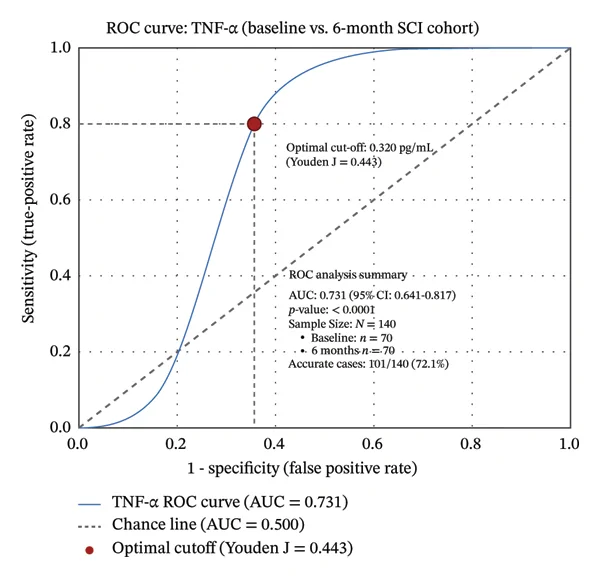
Receiver operating characteristic (ROC) curve for TNF‐α discrimination between baseline and 6‐month measurements in the SCI cohort.

### 3.4. Comparison of Functional Outcomes Between Taurine and Conventional Groups

At baseline, there were no significant differences between the taurine and conventional‐treatment groups in self‐care, respiration and sphincter management, mobility, or total SCIM scores (all *p* > 0.36). At 6 months, however, the taurine group demonstrated substantially greater functional recovery across all SCIM domains.

The mean self‐care score increased to 16.06 ± 3.18 in the taurine group compared with 5.65 ± 2.16 in the conventional‐treatment group (Mann–Whitney *p* < 0.001). Similarly, scores for respiration and sphincter management were higher with taurine (35.11 ± 3.34 vs. 20.41 ± 3.60; *p* < 0.001), as were mobility scores (19.97 ± 5.57 vs. 8.47 ± 2.45; *p* < 0.001).

Consistent with these domain‐specific improvements, the total SCIM score at 6 months was markedly higher in the taurine group than in the conventional‐treatment group (71.14 ± 9.78 vs. 34.53 ± 4.76; *p* < 0.001). The corresponding median total SCIM scores were 74 (IQR, 69–77) and 34 (IQR, 31.25–37) respectively. Thus, despite comparable baseline functional status, patients receiving taurine supplementation showed substantially greater functional recovery across all SCIM domains at 6 months (Table 7, Figure 4).

**TABLE 7:** Comparison of functional subscale scores between taurine and conventional groups.

Outcome	Time	Taurine (*N* = 36)	Conventional (*N* = 34)	*p*
Self‐care	Baseline	0.11 ± 0.32; 0.00 (0.00–0.00)	0.15 ± 0.36; 0.00 (0.00–0.00)	0.663
	6 months	16.06 ± 3.18; 17.00 (15.75–17.00)	5.65 ± 2.16; 6.00 (4.25–6.00)	< 0.001

Respiration and sphincter management	Baseline	10.00 ± 0.00; 10.00 (10.00–10.00)	10.00 ± 0.00; 10.00 (10.00–10.00)	1.000
	6 months	35.11 ± 3.34; 37.00 (33.00–37.00)	20.41 ± 3.60; 22.00 (17.00–22.00)	< 0.001

Mobility	Baseline	0.19 ± 0.40; 0.00 (0.00–0.00)	0.26 ± 0.45; 0.00 (0.00–0.75)	0.492
	6 months	19.97 ± 5.57; 23.00 (15.00–24.00)	8.47 ± 2.45; 9.00 (7.00–10.75)	< 0.001

Total SCIM score	Baseline	10.31 ± 0.47; 10.00 (10.00–11.00)	10.41 ± 0.50; 10.00 (10.00–11.00)	0.361
	6 months	71.14 ± 9.78; 74.00 (69.00–77.00)	34.53 ± 4.76; 34.00 (31.25–37.00)	< 0.001

**FIGURE 4:**
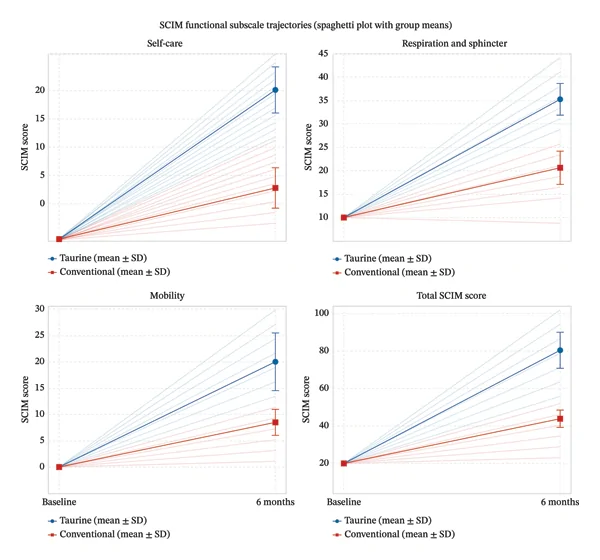
SCIM functional subscale trajectories from baseline to 6 months in the taurine and conventional‐treatment groups.

### 3.5. Longitudinal Association Between TNF‐α and Functional Independence

Higher TNF‐α concentrations were consistently associated with poorer functional status over time. At baseline, TNF‐α was inversely correlated with SCIM total score (*r* = −0.431, 95% CI −0.604 to −0.218; FDR‐adjusted *p* < 0.001) and mobility (*r* = −0.363, 95% CI −0.551 to −0.140; FDR‐adjusted *p* = 0.003), whereas the association with self‐care was not significant (*r* = −0.161, 95% CI −0.381 to 0.077; FDR‐adjusted *p* = 0.183). At 6 months, inverse associations were evident across all evaluable SCIM domains, including total SCIM score (*r* = −0.562, 95% CI −0.704 to −0.377), self‐care (*r* = −0.471, 95% CI −0.636 to −0.266), respiration and sphincter management (*r* = −0.576, 95% CI −0.714 to −0.394) and mobility (*r* = −0.518, 95% CI −0.671 to −0.322; all FDR‐adjusted *p* < 0.001). Repeated‐measures correlation incorporating both baseline and 6‐month observations demonstrated robust inverse longitudinal associations between TNF‐α and SCIM total score (*r*_rm = −0.673, 95% CI −0.784 to −0.522), self‐care (*r*_rm = −0.684, 95% CI −0.791 to −0.536), respiration and sphincter management (*r*_rm = −0.642, 95% CI −0.761 to −0.480) and mobility (*r*_rm = −0.681, 95% CI −0.789 to −0.532; all FDR‐adjusted *p* < 0.001) (Table 8, Figure 5).

**TABLE 8:** Longitudinal associations between TNF‐α and SCIM functional domains in SCI cohort.

SCIM outcome	Baseline *r* (95% CI)	*p*	FDR q	6‐month *r* (95% CI)	*p*	FDR q	Repeated‐measures *r*_rm (95% CI)	*p*	FDR q
SCIM total score	−0.431 (−0.604 to −0.218)	< 0.001	< 0.001	−0.562 (−0.704 to −0.377)	< 0.001	< 0.001	−0.673 (−0.784 to −0.522)	< 0.001	< 0.001
Self‐care	−0.161 (−0.381 to 0.077)	0.183	0.183	−0.471 (−0.636 to −0.266)	< 0.001	< 0.001	−0.684 (−0.791 to −0.536)	< 0.001	< 0.001
Respiration and sphincter management	NA^∗^	NA^∗^	NA^∗^	−0.576 (−0.714 to −0.394)	< 0.001	< 0.001	−0.642 (−0.761 to −0.480)	< 0.001	< 0.001
Mobility	−0.363 (−0.551 to −0.140)	0.002	0.003	−0.518 (−0.671 to −0.322)	< 0.001	< 0.001	−0.681 (−0.789 to −0.532)	< 0.001	< 0.001

*Note:* Values are presented as correlation coefficients (^∗^ *r* ^∗^) with 95% confidence intervals (CIs). ^∗^ *r* ^∗^ represents cross‐sectional correlations, whereas ^∗^r^∗^rm represents repeated‐measures correlations across baseline and 6 months. Negative values indicate inverse associations between TNF‐*α* concentrations and SCIM scores. ^∗^ *p* ^∗^ values are two‐sided, and ^∗^ *q* ^∗^ values represent false discovery rate (FDR)–adjusted ^∗^ *p* ^∗^ values. Statistical significance is indicated by *p* ^∗^ < 0.05, *p* ^∗∗^ < 0.01, and *p* ^∗∗∗^ < 0.001. NA indicates that the baseline correlation was not available.

Abbreviations: CI, confidence interval; FDR, false discovery rate; NA, not available; SCIM, Spinal Cord Independence Measure; TNF‐*α*, tumor necrosis factor‐alpha.

**FIGURE 5:**
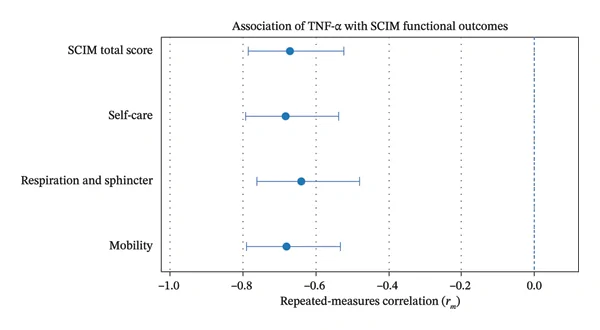
Forest plot showing repeated‐measures correlation coefficients (*r*_*m*_) and 95% confidence intervals for the associations between circulating TNF‐α concentrations and SCIM total score, self‐care, respiration and sphincter management and mobility across baseline and 6‐month assessments. Negative coefficients indicate that higher TNF‐α concentrations were associated with lower functional independence. All longitudinal associations were statistically significant (FDR‐adjusted *p* < 0.001).

### 3.6. TNF‐α–SCIM Associations According to Treatment Group

Repeated‐measures correlation analyses demonstrated inverse associations between TNF‐α concentrations and functional independence across baseline and 6‐month assessments in both treatment groups. In the taurine group, TNF‐α was inversely correlated with SCIM total score (*r*_rm = −0.701), self‐care (*r*_rm = −0.708), respiration and sphincter management (*r*_rm = −0.673) and mobility (*r*_rm = −0.712). Corresponding associations in the conventional‐treatment group were also inverse for SCIM total score (*r*_rm = −0.722), self‐care (*r*_rm = −0.644), respiration and sphincter management (*r*_rm = −0.708) and mobility (*r*_rm = −0.722); all associations were statistically significant after false discovery rate (FDR) correction (adjusted *p* < 0.001).

To formally test whether treatment modified the longitudinal relationship between TNF‐α and functional independence, TNF‐α × time × treatment interactions were evaluated using longitudinal mixed‐effects models. No significant interaction was observed for SCIM total score (*β* = 0.550, 95% CI −0.968 to 2.068; *p*_interaction = 0.478), self‐care (*β* = 0.203, 95% CI −0.780 to 1.187; *p*_interaction = 0.685), respiration and sphincter management (*β* = −0.284, 95% CI −1.647 to 1.079; *p*_interaction = 0.683), or mobility (*β* = 0.247, 95% CI −0.708 to 1.202; *p*_interaction = 0.612). Thus, although higher TNF‐α concentrations were associated with lower SCIM scores in both groups, there was no statistical evidence that taurine supplementation altered the longitudinal strength of the TNF‐α–SCIM association (Table 9).

**TABLE 9:** Longitudinal association of TNF‐α with functional independence and modification by taurine supplementation.

**Outcome**	**r_rm taurine**	**r_rm conventional**	**TNF-α × time × treatment *β* (95% CI)**	**Pinteraction**	**FDR P**

SCIM total score	−0.701	−0.722	0.550 (−0.968–2.068)	0.478	0.685
Self‐care	−0.708	−0.644	0.203 (−0.780–1.187)	0.685	0.685
Respiration and sphincter	−0.673	−0.708	−0.284 (−1.647–1.079)	0.683	0.685
Mobility	−0.712	−0.722	0.247 (−0.708–1.202)	0.612	0.685

**Multiple regression analysis model**					
**Outcome**	**R** ^2^	**Adjusted** **R** ^2^		**Overall** **p**	

Baseline TNF‐α	0.102	0.017		0.320	
Baseline TSS	0.065	−0.024		0.629	
Baseline self‐care	0.040	−0.052		0.852	
Baseline mobility	0.121	0.037		0.213	
6‐month TNF‐α	**0.494**	**0.437**		**< 0.001**	
6‐month TSS	0.091	−0.028		0.634	
6‐month self‐care	0.106	−0.011		0.520	
6‐month Respiration/sphincter	0.099	−0.003		0.458	
6‐month mobility	0.086	−0.034		0.679	

*Note:* Statistical significance was defined as (*p* < 0.05). Bold values indicate statistically significant results. FDR‐adjusted *p* values were used for the longitudinal interaction analyses.

#### 3.6.1. Multivariable Regression Analysis

Multivariable linear regression models were used to examine independent associations of age, sex, ASIA grade and TLISS with TNF‐α and functional outcomes at baseline and 6 months, with baseline outcome measures and baseline TNF‐α additionally included in the corresponding 6‐month models. Model fit varied substantially across outcomes (Table 9). At baseline, the models explained 4.0%–12.1% of the variance, with none reaching statistical significance (overall *p* = 0.213–0.852). At 6 months, the model for TNF‐α demonstrated the strongest explanatory performance (*R* ^2^ = 0.494; adjusted *R* ^2^ = 0.437; overall *p* < 0.001). Baseline TNF‐α was the only independent predictor that remained significant after FDR correction (*β* = 0.582, 95% CI 0.409–0.756; standardised *β* = 0.640; FDR‐adjusted *p* < 0.001). In contrast, the models for 6‐month TSS, self‐care, respiration/sphincter management and mobility explained only 8.6%–10.6% of the variance and were not statistically significant overall (*p* = 0.458–0.679). Variance‐inflation factors were low across models (approximately 1.0–1.5), indicating no evidence of problematic multicollinearity (Table 9).

## 4. Discussion

In this prospective observational study of patients with acute traumatic SCI, serum TNF‐α concentrations decreased significantly over 6 months, with a substantially greater reduction among patients receiving taurine supplementation than among those receiving conventional treatment. At 6 months, TNF‐α concentrations in the taurine group were no longer significantly different from those in healthy controls, whereas concentrations remained significantly elevated in the conventional‐treatment group. Importantly, higher TNF‐α concentrations were consistently associated with lower functional independence, as reflected by SCIM total and domain‐specific scores. However, the absence of a significant TNF‐α × time × treatment interaction indicates that taurine did not significantly modify the longitudinal relationship between TNF‐α and functional independence. Given the nonrandomised treatment allocation, these findings should be interpreted as associations rather than evidence of a causal treatment effect.

The elevated baseline TNF‐α concentrations observed in patients with SCI are biologically consistent with the inflammatory response that follows acute spinal cord trauma. Secondary injury involves activation of resident microglia and recruitment of peripheral immune cells, with subsequent release of pro‐inflammatory mediators that can contribute to neuronal and oligodendroglial injury, axonal dysfunction and tissue degeneration. Previous clinical studies have similarly reported increased circulating inflammatory cytokines following SCI. Davies et al. [7] demonstrated elevated serum cytokines in patients with SCI, supporting the presence of persistent systemic inflammatory activation after traumatic injury. The present findings extend these observations by demonstrating that serum TNF‐α remained higher than healthy‐control concentrations at 6 months in the overall SCI cohort, although the magnitude of persistence differed substantially between treatment groups.

The longitudinal behaviour of TNF‐α was particularly notable. 1n the overall SCI cohort, concentrations declined from 0.7636 pg/mL at baseline to 0.4913 pg/mL at 6 months. The decline was substantially greater in the taurine group, from 0.7002 to 0.2109 pg/mL, whereas the conventional‐treatment group showed only a modest reduction from 0.8308 to 0.7883 pg/mL. Although these differences are compatible with an anti‐inflammatory association of taurine, they cannot establish that taurine itself caused the reduction because treatment was not randomly assigned. Nevertheless, the direction of the findings is consistent with experimental evidence indicating that taurine can attenuate inflammatory responses after SCI. Nakajima and colleagues reported that taurine administration reduced IL‐6, myeloperoxidase, STAT3 activation and COX‐2 expression after experimental SCI and was accompanied by attenuation of motor impairment [8]. Other experimental work has also shown reductions in inflammatory and oxidative‐stress markers, including TNF‐α, following taurine‐containing interventions [9]. These experimental findings provide biological plausibility for the marked reduction in circulating TNF‐α observed in the present study, although extrapolation from experimental models to clinical treatment efficacy remains uncertain.

The diagnostic analysis further supports the potential clinical relevance of TNF‐α. Baseline serum TNF‐α discriminated patients with acute SCI from healthy controls with an AUC of 0.892 (95% CI 0.829–0.946), with 87.14% sensitivity and 88.57% specificity at the optimal threshold. These findings are consistent with previous evidence identifying TNF‐α as an important inflammatory biomarker after SCI. Xu et al. [3] reported the involvement of TNF‐α in SCI‐related neuroinflammation and neuropathic pain, supporting its biological relevance. Similarly, previous biomarker research has highlighted the potential of inflammatory cytokines for characterising SCI‐related pathology [2]. However, the present ROC analysis evaluates discrimination between SCI and healthy controls and should therefore not be interpreted as demonstrating prognostic performance or prediction of neurological recovery.

The association between TNF‐α and functional independence was consistent across the longitudinal assessments. At baseline, higher TNF‐α was associated with lower total SCIM and mobility scores, whereas by 6 months inverse associations were evident across all evaluable SCIM domains. More importantly, repeated‐measures analyses demonstrated robust inverse associations between TNF‐α and total SCIM, self‐care, respiration and sphincter management and mobility, with all FDR‐adjusted *p* values below 0.001. These findings are consistent with the longitudinal observations of Biglari et al. [10], who reported temporal changes in serum TNF‐α after SCI and found that lower TNF‐α concentrations were associated with neurological improvement. Together, these observations support the interpretation that circulating TNF‐α may reflect the inflammatory milieu accompanying functional status after SCI. However, the observational nature of the association does not establish that TNF‐α directly determines functional recovery.

The comparison between treatment groups showed substantially higher SCIM scores at 6 months among patients receiving taurine, including self‐care, respiration and sphincter management, mobility and total SCIM. These functional findings occurred alongside the greater reduction in TNF‐α in the taurine group and are biologically compatible with the hypothesis that attenuation of inflammatory activity may accompany improved functional recovery. Experimental studies have provided supporting mechanistic evidence for taurine‐related attenuation of inflammatory and oxidative pathways after SCI [8, 9]. However, the present clinical data cannot determine whether reduced TNF‐α contributed directly to functional recovery, whether both outcomes reflected other aspects of recovery, or whether unmeasured differences between treatment groups contributed to both findings.

An important finding that moderates this interpretation was the formal treatment‐interaction analysis. Although repeated‐measures correlations between TNF‐α and SCIM outcomes were significant in both the taurine and conventional‐treatment groups, the TNF‐α × time × treatment interaction was not significant for total SCIM or any individual SCIM domain. Thus, differences in correlation coefficients between groups should not be interpreted as evidence that taurine strengthened or otherwise modified the TNF‐α–functional relationship. This distinction is important because correlation within individual treatment groups does not provide a formal test of whether the association differs between groups. The present results instead indicate a consistent inverse relationship between inflammatory burden and functional independence across the cohort, without statistical evidence that taurine altered that relationship.

The persistence of TNF‐α elevation in a proportion of patients is also consistent with previous observations of prolonged inflammatory activity after SCI. Hayes et al. [11] reported persistent elevations of pro‐inflammatory cytokines in individuals with chronic SCI, suggesting sustained immune dysregulation beyond the acute phase. Detloff et al. [12] similarly demonstrated temporal inflammatory responses after SCI in an experimental model, linking cytokine activity with subsequent pathological consequences. The present study extends these observations by following serum TNF‐α through 6 months and demonstrating that its trajectory was associated with functional status. Nevertheless, differences in species, sampling compartments, injury characteristics and disease stage mean that direct quantitative comparisons with experimental studies should be made cautiously.

The multivariable findings provide an additional perspective. Baseline TNF‐α was the only variable that independently predicted 6‐month TNF‐α after adjustment for age, sex, baseline neurological status, injury severity and the relevant baseline outcome measure. The model explained approximately half of the variance in 6‐month TNF‐α (*R* ^2^ = 0.494), whereas the multivariable models for functional outcomes showed limited explanatory performance. These findings suggest that the longitudinal inflammatory trajectory may be partly related to the patient’s initial inflammatory state, while functional recovery is likely determined by multiple interacting biological and clinical factors. Accordingly, TNF‐α should be regarded as a candidate biomarker associated with the recovery process rather than as an isolated determinant of functional outcome.

The findings should be interpreted in light of several limitations. First, the sample size was modest and the study was conducted at a single centre, which limits precision and generalisability. Second, treatment allocation was nonrandomised and influenced by patient willingness and routine clinical management. Consequently, residual confounding and selection bias cannot be excluded, including differences in rehabilitation exposure, nutritional status, socioeconomic circumstances, caregiver support, treatment preferences and other prognostic factors. Although available baseline demographic and neurological characteristics, including AIS grade, injury level, injury severity and baseline SCIM, were compared between groups, detailed treatment‐related variables were not systematically captured, including time from injury to treatment, specific surgical characteristics and rehabilitation intensity or cumulative rehabilitation exposure. These factors may have influenced both inflammatory trajectories and functional recovery and could not therefore be incorporated into the present analyses. Comparable baseline functional scores between groups do not eliminate these potential sources of bias.

The principal strength of this study is the integration of longitudinal inflammatory measurements with objective functional assessment and comparison with healthy controls. The study also combines diagnostic analysis, repeated‐measures correlation, treatment‐interaction modelling and multivariable analysis, providing complementary perspectives on the relationship between TNF‐α and functional recovery. The findings are consistent with previous experimental and clinical evidence implicating inflammatory pathways in SCI while providing preliminary human data on the association between taurine supplementation, TNF‐α modulation and functional outcomes.

Overall, the findings support an association between lower circulating TNF‐α concentrations and greater functional independence after acute traumatic SCI. Taurine supplementation was associated with a substantially greater reduction in TNF‐α and higher 6‐month SCIM scores than conventional treatment, but the nonrandomised design and absence of a significant TNF‐α × time × treatment interaction preclude attribution of these differences specifically to taurine. Future multicentre randomised controlled trials with concealed allocation, standardised rehabilitation and clinical management, adequate sample size, longer follow‐up and broader inflammatory and neurobiological profiling are required to determine whether taurine has a causal therapeutic effect and to establish whether TNF‐α can serve as a clinically useful biomarker for treatment response or recovery.

### 4.1. Strengths of the Study

This study has several important strengths. First, the prospective longitudinal design enabled assessment of changes in serum TNF‐α and functional independence over a clinically relevant 6‐month period rather than relying solely on cross‐sectional measurements. Second, the inclusion of age‐ and sex‐matched healthy controls provided a reference group for evaluating the magnitude and persistence of inflammatory activation after acute traumatic SCI. Third, functional recovery was assessed using the SCIM, allowing evaluation of both overall functional independence and clinically relevant domains including self‐care, respiration and sphincter management and mobility.

A further strength was the use of complementary statistical approaches to examine different aspects of the study question. These included between‐group comparisons of TNF‐α concentrations, longitudinal correlation analyses, repeated‐measures correlation, formal TNF‐α × time × treatment interaction testing, ROC analysis and multivariable regression. The repeated‐measures analyses demonstrated consistent inverse associations between circulating TNF‐α concentrations and functional independence across the 6‐month observation period, while the formal interaction analysis appropriately tested whether taurine supplementation modified this relationship. Importantly, the latter analysis did not demonstrate a statistically significant treatment interaction, providing a more conservative interpretation of the observed associations.

The study also provides clinically relevant preliminary human data linking taurine supplementation with TNF‐α trajectories and functional outcomes after acute SCI. The marked reduction in TNF‐α in the taurine group, together with 6‐month SCIM differences, generates a clinically testable hypothesis regarding the potential role of taurine as an adjunctive intervention. However, because treatment allocation was nonrandomised, these findings should be regarded as hypothesis‐generating rather than confirmatory evidence of efficacy.

## 5. Conclusion

In this prospective observational cohort of patients with acute traumatic SCI, serum TNF‐α concentrations declined over 6 months and were consistently inversely associated with functional independence measured by the SCIM. Patients who received taurine supplementation had a substantially greater reduction in serum TNF‐α and higher 6‐month SCIM scores than those receiving conventional treatment alone. However, the nonrandomised treatment allocation introduces the possibility of selection bias and residual confounding, and the absence of a significant TNF‐α × time × treatment interaction provides no statistical evidence that taurine modified the longitudinal relationship between TNF‐α and functional recovery.

These findings therefore support TNF‐α as a candidate biomarker associated with the inflammatory and functional recovery trajectory after acute SCI and provide preliminary clinical evidence warranting further investigation of taurine as a potential adjunctive intervention. They should not, however, be interpreted as evidence of a causal therapeutic effect. Adequately powered, multicentre randomised controlled trials with concealed allocation, standardised clinical and rehabilitation protocols, rigorous monitoring of adherence and concomitant care, serial inflammatory profiling and longer follow‐up are required to establish the efficacy and safety of taurine supplementation and to determine whether TNF‐α has clinically useful prognostic or treatment‐response value in acute SCI.

### 5.1. Safety Outcomes

Safety was monitored throughout the 6‐month study period. Routine laboratory investigations, including complete blood count (CBC), liver function tests (LFTs) and kidney function tests (KFTs), were performed according to the institutional clinical protocol. No clinically significant laboratory abnormalities attributable to taurine supplementation were observed. No adverse events were reported in any participant during the study period, and no participant discontinued taurine supplementation because of an adverse event or treatment intolerance. Overall, no clinically relevant safety concerns related to taurine supplementation were identified during the study.

## Author Contributions

Zeenat Ara: conceptualisation, methodology, data curation, study design, formal analysis, writing–original draft, visualisation, laboratory work, data collection, data analysis, interpretation of results and drafting of the manuscript.

Shah Waliullah: investigation, supervision, validation, writing–review and editing, critical revision of the manuscript, interpretation of findings and final approval of the manuscript.

## Funding

This research received no external funding.

## Ethics Statement

This study was approved by the Institutional Ethics Committee of King George’s Medical University, Lucknow, India (Approval No: Ref Code No 104th ECM IIB‐Ph.D/P3, Dated 3/02/2021). Written informed consent was obtained from all participants prior to inclusion in the study. The study was conducted in accordance with the Declaration of Helsinki.

## Consent

Please see the Ethics Statement.

## Conflicts of Interest

The authors declare no conflicts of interest.

## Data Availability

The data that support the findings of this study are available on request from the corresponding author. The data are not publicly available due to privacy or ethical restrictions.
